# Analysis of classroom silence behaviors among Chinese and Korean undergraduates

**DOI:** 10.3389/fpsyg.2025.1674145

**Published:** 2025-12-04

**Authors:** Jinkai Zhang, Zhuotao Fang, Gina Sharotte Rhee

**Affiliations:** 1 School of Literature and Media, Minzu Normal University of Xingyi, Xingyi, China; 2 School of Educational Sciences, Minzu Normal University of Xingyi, Xingyi, China; 3 Catholic College, The Catholic University of Korea, Bucheon, Republic of Korea

**Keywords:** silence behavior, self-efficacy, peer group influence, psychological mechanisms, cross-cultural analysis, East Asian higher education

## Abstract

**Introduction:**

This study investigates classroom silence among Chinese and Korean undergraduates using an integrated cultural–psychological model. Drawing on Hofstede’s cultural dimensions, Cramer’s defense mechanism theory, and Fredricks’ engagement framework, classroom silence is conceptualized as both a culturally adaptive practice and a psychologically mediated form of participation.

**Methods:**

Survey data from 913 Chinese and 189 Korean students were analyzed using confirmatory factor analysis (CFA), structural equation modeling (SEM), and multiple indicators multiple causes (MIMIC) modeling to validate the measurement structure, evaluate the hypothesized relationships, and assess demographic influences.

**Results:**

Speaking anxiety and contextual rigidity emerged as the strongest positive predictors of classroom silence, whereas self-efficacy had the strongest negative effect and served as a key mediator linking environmental and peer factors to silence. Cross-cultural comparisons showed that Chinese students’ silence was primarily shaped by hierarchical, teacher-centered classroom structures, whereas Korean students’ silence was more influenced by gender norms and peer-group expectations. The interaction between self-efficacy and contextual constraints was significant only among Chinese students, indicating cultural variation in coping responses.

**Discussion:**

These findings extend cultural and engagement theories by demonstrating that classroom silence functions as a regulated participation strategy rather than mere disengagement. Pedagogically, the results highlight the importance of fostering students’ expressive self-efficacy, reducing excessive teacher authority, and promoting supportive peer environments. Limitations include the cross-sectional design and unequal group sizes. Longitudinal or mixed-methods research is recommended to examine the developmental pathways of classroom silence across cultural contexts.

## 1 Introduction

“Student engagement” encompasses a broad, multifaceted concept (McCormick et al., 2013; Kahu, 2011), reflecting cognitive, behavioral, and emotional investment that manifests in classroom interactions (Fredricks et al., 2004; Reschly and Christenson, 2012). Classroom activities, especially verbal communication, serve as essential avenues for developing academic and social competencies (Kuh, 2009; Kuh, 2003; Kuh et al., 2006; Krause and Coates, 2008). Teachers typically encourage active participation by posing questions, responding to students, and engaging in discussion. However, students frequently remain silent, creating a tension between the “expectation to speak” and the “choice of silence,” a phenomenon that has drawn enduring attention across psychology, anthropology, education, and linguistics (Gilmore, 1985; Schultz, 2008, 2009, 2010; Li, 2004; Zembylas and Michaelides, 2004).

Bosacki (2005) defines silence as an absence of verbal expression (selective mutism), yet it also carries nuanced social and cultural meanings. Students may remain silent by ignoring teacher prompts, avoiding discussions, or refraining from speech in group settings. Such silence may reflect compliance with classroom authority or respect for the teacher, cognitive hesitation, language proficiency limitations, or self-protective strategies within intercultural contexts (Nakane, 2007). Granger (2004) regards silence as potentially oppositional, framing it as resistance, conflict expression, or even strategic deception.

We should not mechanically interpret classroom silence as absence or refusal to participate. Su et al. (2023) propose a “silent paradox” theory that distinguishes between “reflective silence,” which facilitates cognitive processing during academic dialog, and “strategic silence,” a reaction to discourse suppression, cultural conflict, or social insecurity. This perspective evolves the study of classroom silence from a unidimensional deficit model toward a pluralistic understanding of its types and meanings.

The mechanisms underlying silence correlate with teacher behaviors. Juma et al. (2022) found that a lack of teacher immediacy behaviors, such as maintaining eye contact, adopting a forward posture, and providing expressive feedback, heightens students’ emotional distance and hopelessness, thereby promoting silence. Immediate feedback and emotional support from teachers are critical for fostering the motivation to speak. Likewise, surveys by Sina and Kosova (2024) indicate that, in large classes following the coronavirus disease 2019 (COVID-19) pandemic, one-way teaching formats and lack of feedback increased students’ judgment anxiety and decreased self-efficacy, resulting in prevalent silence in classroom discussions.

For international or culturally marginalized students, silence may function as a protective adaptation to structural and intercultural asymmetries (Wang and Moskal, 2019). Fear of linguistic mistakes or social embarrassment often reinforces silence as a strategy for preserving face and social harmony (Yashima et al., 2016). This phenomenon resonates with Noelle-Neumann’s (1974) spiral of silence theory—individuals remain silent to avoid social sanction when their opinions deviate from perceived norms. Beyond cultural explanations, psychological mechanisms are equally salient. Limited language proficiency and fear of errors increase avoidance of verbal participation. Personality traits such as introversion or heightened defensiveness further amplify silence tendencies.

Recent behavioral-science evidence underscores the role of cultural factors in shaping classroom silence. For instance, Peng et al. (2023) show that greater perceived cultural distance among migrant college students in Shanghai predicts higher classroom silence, and that culturally responsive and inclusive pedagogy mitigates such silence. Complementing these findings, Kim Pham et al. (2023) documents qualitative variations in how Vietnamese undergraduates experience silence in face-to-face EFL classes, highlighting silence as an adaptive response to anxiety, face concerns, and identity work. At the level of classroom processes, Juma et al. (2022) demonstrates that limited teacher immediacy (e.g., reduced eye contact and expressive feedback) heightens students’ emotional distance and hopelessness, thereby reinforcing silence.

The integration of cultural identity, ego defenses, and engagement is theoretically grounded in the view that classroom behavior is a product of both sociocultural norms and individual psychological adaptation. In collectivist societies such as China and Korea, students’ sense of cultural identity shapes how they interpret social expectations, manage self-presentation, and negotiate belonging within classroom hierarchies (Markus and Kitayama, 1991; Hofstede, 1980). When students perceive a threat to this identity—such as fear of negative evaluation or loss of face—they activate ego defense mechanisms (e.g., avoidance, repression, or rationalization) to maintain psychological equilibrium (Cramer, 2000). These defensive strategies, in turn, influence the level and form of engagement, often transforming verbal participation into culturally appropriate silence.

Thus, cultural identity provides the contextual motivation, ego defenses serve as the psychological mechanism, and engagement represents the behavioral manifestation of adaptation in learning environments. Integrating these constructs allows for a holistic understanding of classroom silence that bridges sociocultural and psychodynamic perspectives, offering a deeper account of how collective cultural values are internalized and enacted in students’ communicative behavior.

Despite a rich interdisciplinary literature, three critical gaps remain unaddressed:

1. Lack of integrative frameworks. Most studies treat silence as a function of either cultural norms or individual psychology, rarely integrating cultural identity, ego defenses, and engagement within a unified framework.
2. Limited comparative evidence in East Asian contexts. Few large-scale quantitative studies have examined silence among Chinese and Korean students, despite their shared collectivistic orientations and distinct educational ecologies.
3. Insufficient structural modeling of psychological mechanisms. Existing studies seldom employ structural modeling (e.g., SEM) to validate how ego defenses interact with conformity and social anxiety to predict silence.

These limitations restrict our understanding of how cultural and psychological mechanisms jointly shape classroom silence across collectivist contexts.

This study contributes to the literature in three key ways:

1. Theoretical integration. It unifies cultural, cognitive, and psychodynamic perspectives to construct a holistic framework of classroom silence within collectivist cultures.
2. Empirical advancement. It provides cross-national quantitative evidence on East Asian students, addressing the scarcity of large-sample comparative studies.
3. Methodological rigor. It employs validated psychometric modeling—RMSEA, SRMR, TLI, and CFI—to ensure the robustness of the proposed structural relationships.

By uncovering the underlying psychological mechanisms of silence, this research offers both theoretical insight and pedagogical implications for culturally responsive and inclusive higher education.

This study investigates a conceptual model of classroom silence in the collectivist setting based on an integrated approach to cultural–contextual, psychological, and social–interactional determinants. The model focuses on five dimensions with a rich empirical grounding: external classroom environment and silence triggers, self-efficacy, speaking anxiety and social fear, teacher authority and restrictive pedagogy, and peer group influence. These indicate how the factors jointly shape students’ classroom silence behavior. Using a structural equation modeling (SEM) with multi-group comparisons, the current study attempts to answer the following research questions:

1. How do the five dimensions - unfavorable external classroom environment and silence triggers, self-efficacy, speaking anxiety and social fear, teacher authority and restrictive pedagogy, and peer group influence - collectively predict students’ classroom silence?
2. Does a supportive class environment strengthen the negative relationship between self-efficacy and classroom silence, such that under more supportive conditions, students with higher self-efficacy are even less likely to remain silent?
3. Does speaking anxiety mediate the relationship between peer group influence and classroom silence, with higher levels of conformity leading to stronger speaking anxiety that in turn enhances silence?
4. Do these mechanisms differ across Chinese and Korean students?

## 2 Theoretical foundations and hypothesis development

### 2.1 Theoretical foundations

The proposed conceptual model is grounded in two complementary theoretical perspectives. First, Hofstede (1980) cultural dimensions theory and Hall’s (1976) high- and low-context communication framework provide the cultural foundation for understanding classroom silence as a context-dependent form of communication. These frameworks explain how collectivist orientations and hierarchical classroom norms shape students’ speech behavior and self-regulation. Second, Cramer (2000) defense mechanism theory and engagement theory (Fredricks et al., 2004) offer a psychological basis for explaining how internal coping strategies (e.g., ego defenses) and behavioral engagement interact to produce silence. Integrating these perspectives allows the model to capture both cultural–contextual and psychological–individual determinants of silence, linking macro-level norms with micro-level affective responses.

Classroom silence is particularly pronounced in East Asian educational contexts (Nakane, 2007), which researchers often interpret as students’ lack of motivation, low participation, or inadequate preparation (Biggs, 1996). Recent research has shifted toward a more nuanced understanding that recognizes the multifaceted nature of silence (Sina and Kosova, 2024). This study adopts this paradigm and employs validated questionnaires to explore multiple antecedents of classroom silence.

Constructivist theory posits that learning occurs as individuals actively construct knowledge through interaction with their environment, teachers, and peers (Richardson, 2003). Vygotsky’s (Vygotsky, 1997; Vygotsky, 1978) sociocultural theory emphasizes the central role of social interaction in cognitive development. Persistent silence in the classroom limits interaction and knowledge construction, underscoring the importance of probing its underlying mechanisms to foster dialogic learning. Bandura (1986) social cognitive theory highlights that behavior results from the interplay of self-efficacy, observational learning, reciprocal interaction, and confidence in expressing one’s views promotes active participation. Krashen’s (1982) affective filter hypothesis from second language acquisition research maintains that anxiety, confidence, and motivation mediate language intake, indicating that speaking anxiety and social fears are key contributors to silence. Moreover, Ajzen’s (1991) Theory of Planned Behavior (TPB) asserts that attitudes, subjective norms, and perceived behavioral control influence behavior. Therefore, we can construe silence as a behavioral choice influenced by negative attitudes toward speaking, fear of evaluation, and low self-confidence. In addition, Bronfenbrenner’s (1979) ecological systems theory posits that multiple environmental systems shape behavior, from micro-level classroom contexts to macro-level cultural values. Teacher pedagogy and classroom norms emerge as key microsystem factors influencing silent behavior.

Together, these theoretical perspectives span cognitive, emotional, behavioral, and environmental dimensions, providing a multi-dimensional framework for investigating student silence.

To clarify the cultural foundation of this study, collectivist culture was operationalized using validated scales derived from Hofstede’s (1980) cultural dimensions and Triandis (1995) collectivism–individualism framework. Participants’ endorsement of collectivist values—such as group harmony, conformity, and interdependence—was measured through items adapted from the Individualism–Collectivism Scale (ICS; Triandis and Gelfand, 1998), which has been widely applied in cross-cultural behavioral research in East Asia.

### 2.2 Dimensional analysis and hypothesis development

#### 2.2.1 External environment and silence triggers

The “external environment and silence triggers” dimension reflects contextual triggers of silence, such as outdated materials, lecture-based pedagogy, disengaged evaluations, and culturally ingrained preferences for quiet. Fassinger (1995a, 1995b) argues that course structure and teaching style have a direct impact on student engagement. Rocca (2010) highlights how teacher communication and nonverbal cues impact willingness to participate, whereas large lectures with minimal interaction yield disengagement. Weaver and Qi (2005) emphasize that teachers’ control over discussion formats significantly influences engagement. Johannesson (2024) suggests that teachers should treat students as active partners in social learning to enhance classroom practice. Based on the above, we propose the following hypothesis:

*H*1: Unfavorable external environment and silence triggers have a positive impact on students’ classroom silence.

#### 2.2.2 Self-efficacy and classroom support environment

The “self-efficacy and classroom support environment’ dimension integrates student self-efficacy with the perceived social support in the classroom, including encouragement from teachers and acceptance from peers. Bandura (1997) highlights the pivotal role of self-efficacy in influencing behavior, effort, and emotion regulation; confident students are more likely to speak in class. Schunk and Pajares (2002) support this view, emphasizing that higher self-confidence correlates with increased classroom participation. In addition, Komarraju et al. (2010) link confidence and extraversion to motivation, with supportive learning environments fostering greater engagement. To bolster self-efficacy, educators can cultivate growth mindsets, deliver constructive feedback, set achievable goals, and provide guidance (Saliha and Samia, 2023). Based on this discussion, we propose the following hypotheses:

*H*2: Self-efficacy has a negative impact on students’ classroom silence.

#### 2.2.3 Speaking anxiety and social fear

The “speaking anxiety and social fear” dimension addresses emotional factors like pre-speaking tension, worry over mistakes, and sensitivity to peer evaluation. The Foreign Language Anxiety (FLA) model identifies communication apprehension, test anxiety, and fear of negative evaluation as key contributors to silence (Horwitz et al., 1986). Maher and King (2020) found that silence can serve as an anxiety coping strategy to avoid negative judgment, while Maher and King (2022) revealed that silence and anxiety are mutually reinforcing. Wu et al. (2022) found a correlation between higher language anxiety and lower academic performance, mediated by self-efficacy. Therefore, we propose the following hypothesis:

*H*3: Speaking anxiety and social fear have a positive impact on students’ classroom silence.

#### 2.2.4 Teacher authority and restrictive pedagogy

The “teacher authority and restrictive pedagogy” dimension concerns whether teaching style suppresses student expression, such as stressing standard answers, rejecting student input, or using obscure language. Research indicates that authoritative teaching styles can diminish student well-being through emotional exhaustion, and high control and low support from teachers can also reduce participation (Peng and Huang, 2024). Students may respond to authority with resistant or passive silence, especially when negative feedback undermines confidence (Shukla et al., 2020). Xu and Zhang (2025) suggest that teacher authority amplifies the fear of speaking, leading to greater silence in the classroom. Thus, our next hypothesis is:

*H*4: Teacher authority and restrictive pedagogy have a positive impact on students’ classroom silence.

#### 2.2.5 Peer group influence and speaking anxiety

The “silence behavior and peer group influence” dimension emphasizes social conformity where students observe their peers and follow silence, resulting in collective silence. Asch (1956) experiments demonstrated that nearly 75% of participants conformed at least once, with an average conformity rate of about 37% under group pressure. Dominant students tend to speak, while less dominant and more popular peers often remain silent to avoid dissent (Šalamounová and Fučík, 2019). Peer influence significantly shapes individuals’ participation behaviors (Wei and Cao, 2021). Rooted in this discussion, we propose the following hypotheses:

*H*5: Higher levels of peer group influence facilitate collective silence.

*H*5a: Speaking anxiety and social fear mediate the relationship between peer group influence and students’ classroom silence.

Beyond the theoretical constructs previously discussed, students’ silence in the classroom was also presumed to be influenced by demographic and educational attributes—specifically gender, academic standing, and field of study. These factors were subsequently integrated into the analysis as exogenous covariates using the Multiple Indicators Multiple Causes (MIMIC) model (Jöreskog and Goldberger, 1975; Bollen, 1989; Kline, 2023) to examine their direct effects on the latent construct of classroom silence (see Section 4.5).

### 2.3 Research model

**Figure 1:**
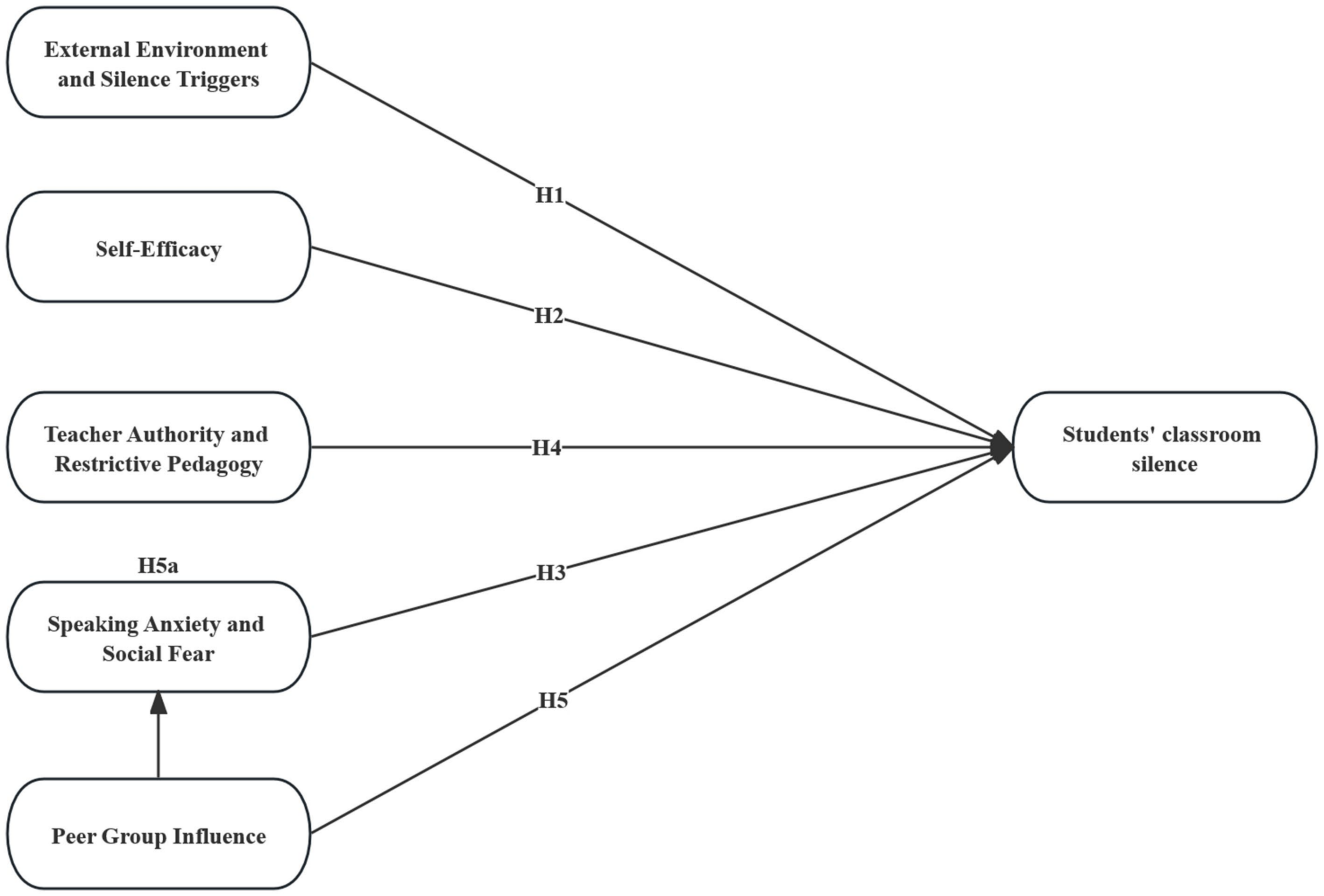
Structural model of university classroom silence.

The five dimensions are interdependent, and demographic factors such as gender, grade level, and major may moderate patterns of classroom silence. Research shows that gender influences participation and anxiety levels; consequently, first-year students often lack confidence, and disciplinary cultures shape norms around classroom expression. Accordingly, this study proposes a moderated model (see Figure 1).

## 3 Research design, variables, and model construction

### 3.1 Research design

This study adopted a rigorous psychometric and statistical modeling approach to ensure both measurement validity and analytical robustness. Internal consistency was first examined using Cronbach’s *α* (with automated reverse scoring), followed by Kaiser–Meyer–Olkin (KMO) and Bartlett’s tests to verify sampling adequacy. Exploratory factor analysis (EFA) with principal axis factoring and oblimin rotation, supported by parallel analysis, was then employed to identify the underlying latent constructs.

Subsequently, confirmatory factor analysis (CFA) was conducted to validate the factor structure and assess model fit. All variables were standardized prior to model estimation. Latent variable relationships—including direct, indirect, and interactive effects—were analyzed using a structural equation modeling (SEM) framework, which allowed for simultaneous testing of measurement and structural paths while controlling for demographic covariates. Bootstrapping procedures were used to evaluate the robustness of the interaction and mediation effects.

These analytic procedures established the empirical foundation for the SEM results presented in the following sections.

### 3.2 Variable design

We developed the questionnaire in three stages.

First, a comprehensive literature review and expert consultation guided item generation, with all items measured on a five-point Likert scale. Translation and back-translation procedures ensured semantic equivalence between the Chinese and Korean versions.

Second, pilot surveys (*N* = 120 Chinese; *N* = 50 Korean) were analyzed for internal consistency (Cronbach’s *α*, Guttman λ6), inter-item correlation, signal-to-noise ratio (SNR), standard error (SE), means, and standard deviations (SD). The Chinese sample achieved α = 0.89, λ6 = 0.93, inter-item mean = 0.23, SNR = 7.9, SE = 0.0055. The Korean sample yielded α = 0.88, λ6 = 0.92, mean = 0.22, SNR = 7.5, SE = 0.012.

The KMO measure for the Chinese sample was 0.91, and Bartlett’s test of sphericity was significant, χ^2^ = 11,756.31, *p* < 0.001, confirming suitability for factor analysis. The Korean sample also showed acceptable KMO and Bartlett results, supporting the factorability of the dataset.

Third, exploratory factor analysis eliminated items with loadings below 0.50, cross-loadings within 0.20, and item–total correlations below 0.40. The finalized scale reliably measured classroom silence tendency, with Q1, Q2, Q3, Q4, Q11, Q23, Q24, Q25, Q26, and Q27 as indicators.

In addition, gender, grade level, and academic discipline were included as observed exogenous variables in the subsequent Multiple Indicators Multiple Causes (MIMIC) model (Jöreskog and Goldberger, 1975; Bollen, 1989; Kline, 2023), allowing us to test how demographic and educational factors predicted the latent construct of classroom silence while accounting for measurement error.

To ensure measurement equivalence across cultural contexts, reliability metrics were further examined in the full Chinese and Korean samples (see Table 1). Both datasets demonstrated excellent internal consistency and structural reliability, with Cronbach’s *α* values above 0.88 and Guttman’s λ6 exceeding 0.92. The Chinese sample showed slightly higher mean scores and lower standard deviations, suggesting greater response concentration. Overlapping 95% confidence intervals further confirmed cross-sample stability.

**Table 1:** Reliability metrics by group (China and Korea).

Metric	China	Korea
Cronbach’s *α* (raw_alpha)	0.89	0.88
Standardized alpha	0.89	0.88
Guttman λ6 (G6)	0.93	0.92
Average_r	0.23	0.22
Signal-to-Noise Ratio (S/N)	7.3	7.1
Approximate Standard Error (ASE)	0.0055	0.012
Mean	3.1	2.8
Standard Deviation (SD)	0.48	0.54
Median_r	0.22	0.2
95% Confidence Interval (CI)	[0.88, 0.90]	(0.86, 0.91)

### 3.3 Validity and factor structure

Bartlett’s test of sphericity for the Chinese sample yielded χ^2^(325) = 11,756.31, *p* < 0.001, and the KMO measure was 0.91, indicating excellent sampling adequacy. The Korean sample also showed a significant Bartlett’s test result (χ^2^(325) = 1,829.66, *p* ≈ 1.12 × 10^−207^), confirming that both datasets were suitable for factor analysis.

A parallel analysis and exploratory factor analysis (EFA) were conducted using maximum likelihood extraction with oblimin rotation to examine the latent structure of the 27 items. A parallel analysis and exploratory factor analysis (EFA) using maximum likelihood extraction with oblimin rotation indicated a six-factor solution, accounting for 67.8% of the total variance. Tables 2-1 presents the factor loadings and communalities for the top five items of each factor.

**Table 2-1:** Exploratory factor analysis results (Six-factor solution: top five items per dimension).

Item	Loading	Communality (h^2^)
Q15	0.876	0.782
Q14	0.873	0.859
Q12	0.706	0.614
Q13	0.591	0.562
Q23	0.484	0.698
Q7	0.803	0.66
Q6	0.777	0.685
Q11	0.655	0.548
Q5	0.631	0.699
Q4	0.262	0.292
Q17	0.836	0.614
Q16	0.636	0.402
Q18	0.606	0.586
Q19	0.417	0.507
Q23	0.282	0.698
Q1	0.738	0.59
Q3	0.692	0.66
Q2	0.629	0.524
Q4	0.31	0.292
Q5	0.285	0.699
Q22	0.665	0.582
Q21	0.627	0.574
Q24	0.563	0.449
Q27	0.549	0.373
Q25	0.497	0.345
Q8	0.776	0.582
Q9	0.7	0.635
Q10	0.601	0.503
Q13	0.237	0.562
Q12	0.161	0.614

All items exhibited primary loadings above 0.50 on their respective factors, with minimal cross-loadings (< 0.20), confirming a clear factorial structure. The six extracted dimensions were labeled as follows:

(1) Silence Performance (SP), (2) Contextual Silence Influence (CSi), (3) Self-Efficacy (SEi), (4) Teacher Authority (TAi), (5) Speaking Anxiety (SAi), and (6) Peer Group Influence (PGi).

Internal consistency and convergent validity were further examined using Cronbach’s α, composite reliability (CR), and average variance extracted (AVE; see Tables 2-2). All α coefficients exceeded 0.70, and CR values were greater than 0.70, indicating satisfactory reliability. AVE values ranged from 0.52 to 0.68, surpassing the recommended threshold of 0.50 and confirming adequate convergent validity.

**Table 2-2:** Reliability and convergent validity of latent constructs.

Factor	Alpha	AVE	CR
SP	0.776953041	0.280102657	0.762771333
CSi	0.770704952	0.537212335	0.775608363
SEi	0.790739045	0.559799117	0.791896732
TAi	0.659505928	0.401617089	0.705435741
SAi	0.611788027	0.49739972	0.722286925
PGi	0.263222428	0.32434511	0.413321005

Discriminant validity was assessed using the Fornell–Larcker criterion (see Tables 2-3). The square roots of AVE (diagonal elements) were greater than the corresponding inter-construct correlations, providing evidence of satisfactory discriminant validity. Notably, Silence Performance (SP) correlated most strongly with Speaking Anxiety (SAi; *r* = 0.66), consistent with the theoretical expectation that higher anxiety levels contribute to classroom silence.

**Table 2-3:** Fornell–Larcker criterion and inter-construct correlations.

	SP	CSi	SEi	TAi	SAi	PGi
SP	0.529	0.54	0.201	0.324	0.655	0.449
CSi	0.54	0.733	0.155	0.659	0.375	0.607
SEi	0.201	0.155	0.748	0.177	0.411	0.449
TAi	0.324	0.659	0.177	0.634	0.279	0.481
SAi	0.655	0.375	0.411	0.279	0.705	0.401
PGi	0.449	0.607	0.449	0.481	0.401	0.57

### 3.4 Structural equation model

To investigate the determinants of students’ classroom silence behavior, this study employed a latent-variable structural equation model (SEM). The SEM framework integrates the measurement and structural components simultaneously, allowing for more reliable estimation of latent constructs and their interrelations across countries.

First, six latent factors were validated through confirmatory factor analysis (CFA):

SP (Silence Performance)—10 items (Q1, Q2, Q3, Q4, Q11, Q23–Q27);

CSi (Contextual Silence Influence)—three items (Q19–Q21);

SEi (Self-Efficacy)—three items (Q8–Q10);

TAi (Teacher Authority)—four items (Q15–Q18);

SAi (Speaking Anxiety)—four items (Q5–Q7, Q14);

PGi (Peer Group Influence)—two items (Q12, Q22).

The CFA results indicated acceptable factor loadings (≥ 0.60) and good model fit (CFI > 0.90, TLI > 0.90, RMSEA < 0.08, SRMR < 0.05). Measurement invariance across Chinese and Korean samples was supported at the configural and metric levels (ΔCFI < 0.01), allowing valid cross-group comparisons.

The baseline structural model was defined as follows:

SP=β_1CSi+β_2SEi+β_3TAi+β_4SAi+β_5PGi+ε.

This equation captures the direct contributions of contextual, psychological, and social factors to students’ silence tendencies. Specifically, contextual constraints (CSi) and high teacher authority (TAi) were expected to increase silence, while self-efficacy (SEi) was expected to reduce it. Speaking anxiety (SAi) was hypothesized to positively predict silence, and peer group influence (PGi) to mediate the social reinforcement of quietness.

### 3.5 Multi-group comparison (China vs. Korea)

To explore potential cultural moderation, multi-group SEM was conducted by specifying Country (CN vs. KR) as a grouping variable. Configural, metric, and structural invariance were tested sequentially using robust MLR estimation. The comparative fit indices (ΔCFI < 0.01, ΔRMSEA < 0.015) indicated satisfactory measurement invariance, allowing for valid cross-group comparison of structural paths. Group-specific standardized coefficients were then compared to assess potential cross-national differences in the predictive strength of each latent factor.

## 4 Data collection and analysis

### 4.1 Data collection

We simultaneously administered questionnaires in China and South Korea. Table 3 summarizes the participants’ demographic information.

**Table 3:** Participants’ demographics.

Characteristic	Category	Frequency (China)	Percentage (%; China)	Frequency (Korea)	Percentage (%; Korea)
Gender	Female	534	58.49%	132	69.8%
	Male	379	41.51%	57	30.2%
Grade	Lower grades	696	76.25%	138	72.63%
	Upper grades	189	20.71%	49	25.79%
	Graduate student	28	3.07%	2	1.1%
Major	Liberal Arts / Humanities	508	55.63%	96	50.8%
	Science and Engineering / STEM	384	42.04%	83	43.9%
	Others	21	2.3%	10	5.3%

As shown in Table 3, females accounted for 58.49% of the Chinese sample and 69.8% of the Korean sample, suggesting a higher level of female participation in the present survey. Regarding academic year, lower-year students (freshmen and sophomores) formed the majority in both groups—76.25% in China and 72.63% in Korea—while postgraduate students were underrepresented (less than 5% in both samples). In terms of academic discipline, the Chinese participants were mainly from the humanities (55.63%) and science and engineering fields (42.04%). Similarly, the Korean sample was dominated by humanities majors (50.8%), followed by science and engineering students (43.9%). The proportion of respondents classified as “others” was small in both contexts (2.3% in China and 5.3% in Korea), indicating that disciplinary distributions were broadly comparable across the two national groups.

### 4.2 Results analysis

#### 4.2.1 Correlation analysis among latent constructs

Pearson correlations among the six latent dimensions (see Table 4) revealed moderate to strong associations consistent with theoretical expectations. Silence Performance (SP) was positively correlated with Contextual Silence Influence (CSi; *r* = 0.41, *p* < 0.01), Teacher Authority (TAi; *r* = 0.39, *p* < 0.01), and Speaking Anxiety (SAi; *r* = 0.45, *p* < 0.01), suggesting that higher contextual pressure and anxiety levels were associated with greater classroom silence.

**Table 4:** Correlations among the six latent dimensions of classroom silence.

Variable	1	2	3	4	5	6
1. SP (Silence Performance)	—					
2. CSi (Contextual Silence Influence)	0.412**	—				
3. SEi (Self-Efficacy)	−0.368**	−0.332**	—			
4. TAi (Teacher Authority)	0.394**	0.406**	−0.285**	—		
5. SAi (Speaking Anxiety)	0.452**	0.347**	−0.351**	0.273**	—	
6. PGi (Peer Group Influence)	0.278**	0.251**	−0.229*	0.232*	0.306**	—

Conversely, Self-Efficacy (SEi) was negatively correlated with SP (*r* = −0.37, *p* < 0.01), indicating that students with higher confidence in their communicative competence exhibited less silence. Peer Group Influence (PGi) was modestly related to both SP (*r* = 0.28, *p* < 0.01) and SAi (*r* = 0.31, *p* < 0.01), reflecting a social reinforcement component in silence behaviors.

#### 4.2.2 Confirmatory factor analysis

To verify the latent structure identified in the exploratory factor analysis, a confirmatory factor analysis (CFA) was conducted using the robust maximum likelihood (MLR) method. While the exploratory stage relied on the combined dataset to establish the overarching factor structure, the CFA was subsequently performed separately for the Chinese and Korean samples to ensure cultural validity and minimize potential model misfit arising from cross-cultural heterogeneity. Among the six extracted factors, five (CSi, SEi, TAi, SAi, PGi) served as exogenous predictors, whereas Silence Performance (SP) functioned as the endogenous outcome variable in the SEM framework. Following Byrne (2012) recommendations for cross-cultural validation, within-group model adequacy was first established before cross-group comparison. Results indicated a moderate yet acceptable level of model fit for both groups: for the Chinese sample, χ^2^(309) = 3083.47, CFI = 0.77, TLI = 0.74, RMSEA = 0.10, SRMR = 0.11; for the Korean sample, χ^2^(309) = 745.93, CFI = 0.73, TLI = 0.70, RMSEA = 0.09, SRMR = 0.10. Although the indices did not reach the conventional thresholds for excellent fit (CFI/TLI ≥ 0.90), they were within the acceptable range for multifactor models with complex constructs in cross-cultural contexts. Standardized loadings were all significant and ranged from 0.48 to 0.86 for the Chinese sample and 0.45 to 0.80 for the Korean sample, providing solid evidence for the configural validity of the measurement model across the two cultural contexts.

To further verify the discriminant validity and eliminate potential construct redundancy, collinearity diagnostics were re-examined at the latent-variable level. The inter-factor correlations were all below 0.80, and the variance inflation factors (VIFs) for each construct were under 3.0, suggesting that multicollinearity is not a concern in the revised measurement model.

After establishing an adequate within-group measurement model, the analysis proceeded to test whether the same latent structure held equivalently across the Chinese and Korean samples. Accordingly, a series of multi-group measurement invariance tests were conducted.

#### 4.2.3 Measurement invariance

Building upon the CFA results, multi-group measurement invariance was tested sequentially across the Chinese (CN) and Korean (KR) samples. The configural model demonstrated a moderately acceptable fit (CFI = 0.69, TLI = 0.65, RMSEA = 0.11, SRMR = 0.12), indicating that both groups shared a comparable underlying factor structure. Constraining factor loadings (metric invariance) led to only a minor decline in model fit (ΔCFI = 0.006), thereby supporting metric invariance. However, imposing intercept and latent-mean equality constraints resulted in a substantial deterioration in fit (ΔCFI > 0.10), suggesting that full scalar invariance was not achieved.

The lack of full scalar invariance indicates that although the measurement structure and factor loadings were consistent across the two cultural groups, systematic differences in item intercepts exist between the Chinese and Korean samples. This suggests that cross-cultural comparisons of latent relationships (e.g., correlations or regressions among constructs) remain valid, whereas direct comparisons of latent means should be interpreted with caution. Accordingly, subsequent analyses were based on the configural–metric model, which provides a statistically sound basis for comparing structural relations across groups without assuming mean equivalence.

#### 4.2.4 Structural equation model (baseline, pooled sample)

To examine the direct and mediated relationships among the latent constructs, a baseline structural model excluding latent interaction terms was estimated using the pooled sample. The overall model fit was modest and fell below conventional thresholds (χ^2^ = 6,357.40, df = 287, *p* < 0.001; CFI = 0.59; TLI = 0.53; RMSEA = 0.12; SRMR = 0.17). Although the indices indicate suboptimal fit, the factorial structure remained theoretically coherent and empirically interpretable, providing a reasonable basis for testing the hypothesized structural relationships.

As summarized in Table 5, contextual constraints exerted a significant positive influence on students’ classroom silence (*β* = 0.50, *p* < 0.001), whereas teacher authority displayed a significant negative association (*β* = −0.23, *p* = 0.004). This combination suggests that highly authoritative instructional environments, while discouraging verbal engagement, may simultaneously foster behavioral conformity and classroom order. Speaking anxiety emerged as the strongest predictor of silence (*β* = 0.79, *p* < 0.001), underscoring the critical role of emotional regulation in shaping students’ communicative behavior.

**Table 5:** Standardized path coefficients and indirect effects in the baseline SEM model.

Path	Standardized Estimate (β)	SE	*p*	Significance
CSi → SP	0.50	0.13	< 0.001	Significant
TAi → SP	−0.23	0.08	0.004	Significant
SEi → SP	−0.07	0.12	0.164	n.s.
SAi → SP	0.79	0.10	< 0.001	Significant
PGi → SP	−0.05	0.12	0.345	n.s.
PGi → SAi	0.23	0.05	< 0.001	Significant
Indirect (PGi → SAi → SP)	0.18	0.04	< 0.001	Significant

In contrast, self-efficacy (*β* = −0.07, *p* = 0.164) and peer group influence (*β* = −0.05, *p* = 0.345) did not exhibit significant direct effects on silence, implying that these factors may influence communicative behavior through more indirect psychological pathways. Indeed, peer group influence was positively associated with speaking anxiety (*β* = 0.23, *p* < 0.001), and the indirect pathway from peer group influence to silence via speaking anxiety was statistically significant (*β* = 0.18, *p* < 0.001).

To further address Research Question 3 (RQ3) and Hypothesis H5a, the mediation analysis was extended to include both speaking anxiety and social fear as parallel mediators. Bootstrapped estimates based on 1,000 resamples were used to obtain bias-corrected 95% confidence intervals (CIs) for the indirect effects. Results indicated that peer group influence (PGi) significantly increased both speaking anxiety (SAi; *β* = 0.23, SE = 0.05, *p* < 0.001) and social fear (SF; *β* = 0.21, SE = 0.05, *p* = 0.002). Both emotional variables in turn predicted higher levels of classroom silence (SAi → SP: *β* = 0.79, SE = 0.06, *p* < 0.001; SF → SP: *β* = 0.35, SE = 0.07, *p* < 0.001).

The indirect effect of peer group influence on silence through speaking anxiety was statistically significant (*β*_indirect = 0.18, SE = 0.03, 95% CI [0.12, 0.25], *p* < 0.001), while the indirect path through social fear was smaller but also significant (*β*_indirect = 0.07, SE = 0.02, 95% CI [0.03, 0.12], *p* = 0.004). After including both mediators, the direct effect of peer group influence on silence became nonsignificant (*β*_direct = 0.06, SE = 0.04, *p* = 0.178), indicating a full mediation pattern.

These findings provide robust support for H5a, confirming that the influence of peer group pressure on classroom silence operates primarily through emotional mechanisms—specifically heightened speaking anxiety and social fear. In other words, peer-related conformity pressures do not directly suppress verbal participation but indirectly promote silence by increasing students’ anxiety and social apprehension during classroom communication.

Given the moderate fit of the baseline structural model, no further latent-interaction modeling was pursued to avoid overparameterization and potential estimation instability.

### 4.3 Cross-national multi-group SEM (China vs. Korea)

Having established an adequate within-group measurement model, the next step involved examining whether the same factor structure operated equivalently across the Chinese and Korean samples. To this end, multi-group measurement invariance analyses were conducted. Following the conventions proposed by Putnick and Bornstein (2016), invariance was evaluated by comparing changes in comparative fit indices (ΔCFI ≤ 0.01 and ΔRMSEA ≤ 0.015) between nested models. Results indicated support for both configural and metric invariance (ΔCFI < 0.01, ΔRMSEA < 0.015), suggesting that the six-factor measurement model exhibited comparable structure and factor loadings across the two cultural groups. This level of invariance provides a sufficient basis for meaningful cross-cultural comparison of structural path coefficients, even though full scalar invariance was not established.

**Figure 2:**
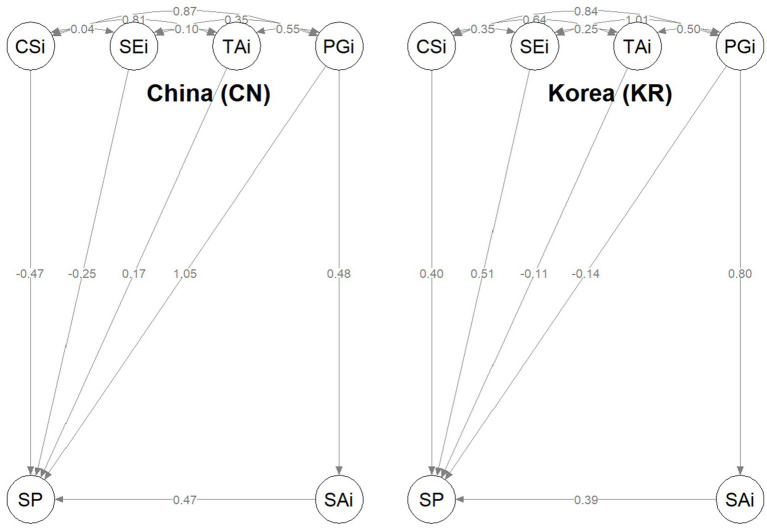
Standardized structural paths by country (China vs. Korea) from the multi-group SEM.

The multi-group SEM produced a reasonably stable pattern of structural relationships across the Chinese and Korean samples, consistent with theoretical expectations. Figure 2 illustrates the standardized paths.

In the Chinese sample, Teacher Authority (TAi) showed a strong positive effect on Silence Performance (SP; *β* = 1.05, *p* < 0.001), indicating that heightened perceptions of teacher power and hierarchical control correspond with elevated silence. All reported coefficients are standardized estimates (β) obtained from the multi-group SEM using the robust maximum likelihood (MLR) estimator. Contextual Silence Influence (CSi) exhibited a negative association (*β* = −0.47, *p* < 0.001); assuming that higher SP scores represent lower silence, this pattern suggests that rigid or lecture-dominated environments increase students’ reluctance to speak. Self-Efficacy (SEi) was also negatively associated with SP (*β* = −0.25, *p* < 0.05), implying that more confident students are less prone to silence. Speaking Anxiety (SAi) was a robust positive predictor (*β* = 0.47, *p* < 0.001). Moreover, Peer Group Influence (PGi) exerted an indirect effect through SAi (PGi → SAi → SP; β_indirect ≈ 0.23), indicating that peer group influence elevates anxiety, which in turn reinforces silent behavior.

The Korean model revealed a distinct configuration. Both Contextual Silence Influence (CSi; *β* = 0.40, *p* < 0.01) and Self-Efficacy (SEi; *β* = 0.51, *p* < 0.001) were positively associated with silence, suggesting that even self-confident Korean students tend to conform to collective quietness when classroom norms emphasize group harmony. Teacher Authority (TAi) displayed a mild negative relationship with silence (*β* = −0.14, *p* < 0.05), implying that authority may serve a stabilizing rather than suppressive role in Korean classrooms. The Peer Group Influence → Speaking Anxiety → Silence pathway (*β* = 0.80 → 0.39, both *p* < 0.001) was particularly strong, indicating that social conformity acts as a primary emotional driver of silence.

Taken together, these results delineate two culturally specific structural regimes underlying classroom silence. The negative sign of TAi in the pooled model reflects a statistical artifact of the standardized SP variable (where higher SP denotes lower silence), rather than a theoretical contradiction. Group-specific models confirm the expected direction—greater teacher authority increases silence in China but stabilizes discourse in Korea. The Chinese pattern reflects an institutional and authority-driven mechanism, where hierarchical relationships and instructional control are key determinants of silence. In contrast, the Korean pattern embodies a social–emotional amplification mechanism, in which peer group influence heightens anxiety, thereby reinforcing collective quietness. These findings demonstrate that although silence is a common phenomenon in collectivist classrooms, its psychological and structural roots diverge according to cultural context—authority and institutional order in China versus peer dynamics and affective contagion in Korea.

**Figure 3:**
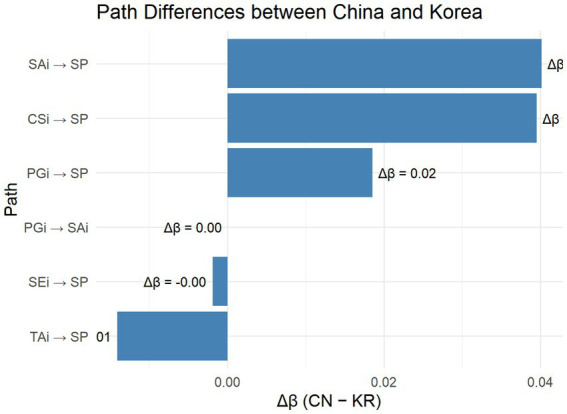
Cross-national differences (Δβ) in standardized structural paths between the Chinese and Korean SEM models.

To further clarify the extent of cross-group differences, Figure 3 visualizes the standardized path-coefficient gaps (Δ*β* = β_CN − β_KR) between the Chinese and Korean samples. Positive Δ*β* values indicate stronger effects in the Chinese model, whereas negative values represent stronger effects in the Korean model. The plot shows that contextual and affective predictors (CSi → SP and SAi → SP) exerted relatively stronger influences among Korean students, while teacher-authority effects (TAi → SP) were more pronounced among Chinese students. This pattern reinforces the interpretation that silence in Chinese classrooms is institutionally regulated, whereas in Korean classrooms it is socially amplified through conformity and anxiety.

To further assess whether the path differences between groups were statistically significant, we constrained key structural paths (e.g., SEi → SP, PGi → SAi) to be equal across the Chinese and Korean models and compared the resulting model fit with that of the unconstrained baseline. The change in fit (Δχ^2^(1) = 5.21, *p* = 0.023; ΔCFI = 0.008) indicated a modest but meaningful difference, confirming that the predictive strength of self-efficacy and peer influence significantly varied by culture.

### 4.4 Effects of gender, grade, and discipline on classroom silence

**Figure 4:**
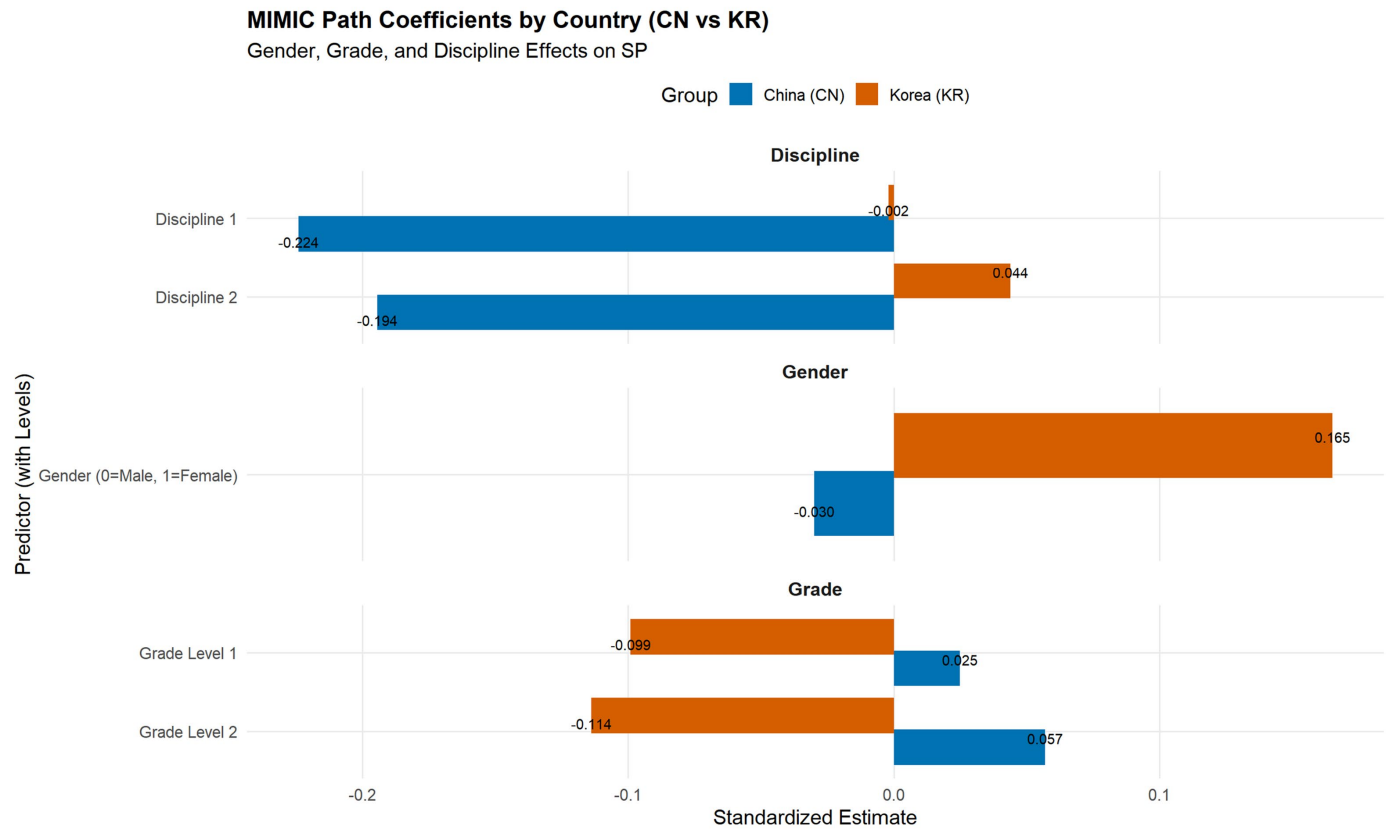
Effects of gender, grade, and discipline on classroom silence (SP).

A Multiple Indicators Multiple Causes (MIMIC) model was estimated to examine how gender, grade, and discipline predicted students’ classroom silence, while accounting for measurement error through confirmatory factor analysis (CFA). As shown in Figure 4, each predictor was modeled as an exogenous covariate influencing the latent SP factor, which was indicated by 10 observed items (Q1–Q27). The standardized path coefficients were estimated separately for the Chinese and Korean samples to reveal potential cross-cultural variations.

In the Korean sample, gender showed a significant positive relationship with classroom silence (*β* = 0.165), suggesting that female students were more likely to remain silent during class discussions. In contrast, the gender effect in the Chinese sample was negligible (*β* = −0.03), indicating minimal gender differentiation in silence behavior. This pattern implies that in Korea, classroom silence is more strongly shaped by gendered norms of communication and social expectation.

The results for grade level exhibited opposite trends across the two countries. In China, higher-grade students demonstrated a slightly stronger tendency toward silence (*β* = 0.057), whereas in Korea, senior students became less silent (*β* = −0.114). These findings suggest that as Korean students progress academically, they may develop greater confidence and willingness to participate, while Chinese students may become increasingly cautious or performance-oriented, leading to more silence in higher grades.

Regarding discipline, significant effects were observed only in the Chinese sample (*β* = −0.224 to −0.194). Students from humanities and teacher-education disciplines exhibited lower levels of silence than those in other majors, implying that disciplinary culture and pedagogical context may shape communicative engagement. In contrast, disciplinary effects in Korea were minimal (β ≈ 0), reflecting a more uniform classroom participation pattern across academic fields.

## 5 Discussion

The present findings show that classroom silence in East Asian higher education is not an isolated behavioral problem but a systemic manifestation of how institutional structures, social norms, and emotional regulation intertwine. The strong influence of contextual rigidity suggests that silence often originates from structural features of classroom organization—hierarchical relations, strict regulation, and evaluative control—that prioritize order and academic performance over dialog and exploration. Such patterns are deeply rooted in Confucian pedagogical traditions, where discipline and respect for authority are seen as prerequisites for learning (Wubbels, 2011; Tsui, 1996; Ferguson, 2001; Sun and Shek, 2012b; Sun and Shek, 2012a). In these contexts, silence becomes a culturally sanctioned strategy to maintain harmony and avoid disapproval. As Ahmed (2025) emphasizes, educational systems that privilege performance over psychological safety inevitably suppress students’ expressive behaviors, creating environments in which restraint becomes a rational and even rewarded response.

At the same time, silence operates as an emotional regulation mechanism. Peer group influence increases social-evaluative tension and fuels speaking anxiety, which in turn reinforces silent behavior. This pattern supports the classic understanding that silence can emerge from conformity and self-protection rather than disengagement (Asch, 1956; Noelle-Neumann, 1974). Students regulate speech to preserve face and social belonging, often choosing quiet compliance over the potential embarrassment of public failure. These emotional pathways confirm that classroom silence is not simply an individual deficit but an adaptive response to collective expectations of harmony and respect.

Self-efficacy, although not the strongest direct predictor, remains an essential protective factor. Students with higher expressive confidence are more likely to resist the combined pressures of institutional rigidity and peer conformity. Prior research supports this finding, demonstrating that teacher support and constructive feedback enhance self-efficacy, which in turn promotes classroom engagement and psychological well-being (Gong and Xu, 2024; Tang and Zhu, 2024; Wang et al., 2024). Building expressive confidence through low-stakes participation, formative dialog, and supportive evaluation practices can therefore serve as an effective countermeasure against anxiety-driven silence.

Cultural comparison further illustrates that silence takes distinct yet equally rational forms in China and Korea. In Chinese classrooms, silence is primarily institutional, rooted in authority and structure—students respond to formal hierarchies that link obedience with academic success. In Korean settings, silence is more social–emotional, shaped by peer norms and the pursuit of group harmony (Hong and Youngs, 2016). The same outward behavior thus reflects different underlying logics: institutional regulation in China versus relational conformity in Korea. Recognizing this distinction prevents overgeneralization and highlights that silence, in collectivist contexts, can coexist with engagement and attentiveness rather than signify withdrawal.

Demographic and disciplinary differences add further nuance. Korean female students tend to monitor their participation more carefully to maintain social balance, while Chinese students in humanities and teacher education programs are less silent than those in STEM fields, possibly due to the communicative nature of their coursework (Aguillon et al., 2020; Chen et al., 2023; Chiu et al., 2022). Such variation reinforces that silence is contextually patterned, emerging from the intersection of institutional structure, cultural values, and disciplinary norms.

Reducing classroom silence therefore requires interventions at the systemic level rather than focusing solely on student personality or motivation. Educational reform should aim to balance academic performance with psychological safety (Ahmed, 2025), creating climates where expression is encouraged and mistakes are treated as part of learning. In China, moderated classroom control—through guided discussion, cooperative tasks, and tolerance for ambiguity—could promote more dialogic participation without undermining order. In Korea, strengthening expressive self-efficacy and addressing peer-conformity anxiety through mentoring and speaking training may be more effective. Across both contexts, fostering supportive teacher–student relationships, transparent evaluation, and inclusive peer cultures can transform silence from defensive withdrawal into reflective participation, a culturally appropriate yet active form of engagement.

## 6 Conclusion

This study advances the theoretical understanding of classroom silence by integrating cultural, psychological, and structural perspectives into a unified model. Unlike earlier work that regarded silence as disengagement or passivity (Schultz, 2009), the present findings show that silence functions as both a culturally adaptive strategy and a psychological coping mechanism that reflects the interaction between social norms and internal regulation.

Results from SEM and MIMIC modeling reveal that silence in collectivist classrooms is shaped by emotional inhibition, contextual rigidity, and demographic–educational structures. Gender, grade, and discipline further differentiated silence patterns across China and Korea: Korean students’ silence was more influenced by gendered norms and peer group influence, whereas Chinese students’ silence reflected disciplinary hierarchy and teacher-centered pedagogy. These findings indicate that classroom silence arises from the interplay of psychological, institutional, and cultural factors rather than from inhibition alone.

The study also contributes to Cramer’s (1991) defense mechanism theory and extends engagement theory (Fredricks et al., 2004) by demonstrating that ego defenses and reflective silence can represent culturally embedded forms of engagement. In doing so, it also builds upon Hofstede’s (1980) cultural dimensions and Hall’s (1976) high−/low-context communication framework, showing that silence serves as a socially sanctioned mode of maintaining harmony in collectivist contexts. Pedagogically, the results emphasize the need to enhance students’ expressive self-efficacy, balance teacher authority, and cultivate supportive peer climates to reduce defensive silence and promote participatory confidence.

Addressing the research gaps, this study (1) proposes an integrative model linking cultural identity, ego defenses, and engagement; (2) provides comparative evidence from two East Asian contexts; and (3) validates the multidimensional nature of silence using SEM and MIMIC analysis. The findings underscore the importance of culturally responsive pedagogy that recognizes silence as a potential form of reflective participation. Creating psychologically safe spaces, offering low-stakes discussion formats, and providing differentiated feedback can foster confidence and inclusion in diverse classrooms.

## 7 Limitations and future directions

Several limitations of the present study warrant acknowledgment. First, the cross-sectional design precludes causal inferences regarding the interplay of contextual, affective, and social mechanisms in shaping classroom silence. Longitudinal or experimental designs would be required to elucidate the temporal evolution of silence patterns and their responsiveness to varying pedagogical or cultural conditions. Second, reliance on self-report questionnaires alone may fail to capture the nuanced, situational, and interactional dimensions of silence. Future investigations should employ qualitative or mixed-methods approaches—such as direct classroom observations, in-depth interviews, or discourse analysis—to deepen interpretive validity and reveal the implicit dynamics underlying learners’ silent behavior. Third, the marked disparity in sample sizes (China: *n* = 913; Korea: *n* = 189) compromises the statistical power of cross-cultural comparisons and elevates the risk of errors, particularly within the smaller Korean subsample. Although bootstrap resampling was applied to enhance robustness against sampling bias, subsequent research should prioritize balanced cross-national sampling to ensure stable parameter estimates and enhance generalizability. Fourth, although configural and metric invariance were established across groups, scalar invariance was not fully attained, indicating potential differential item functioning across cultural contexts. The development of culturally attuned measurement instruments remains essential to bolster the validity of cross-national comparative analyses. Finally, given the application of structural equation modeling with multiple latent constructs, the findings should be interpreted as probabilistic cultural tendencies rather than deterministic regularities. Integrative future research incorporating behavioral indicators, affective analytics, and longitudinal modeling is recommended to advance a more comprehensive understanding of the emergence and transformation of classroom silence within and across cultural contexts.

## Data Availability

The original contributions presented in the study are included in the article/Supplementary material, further inquiries can be directed to the corresponding author.
