# Supplementary material for: Analysis of classroom silence behaviors among Chinese and Korean undergraduates
Source: Front Psychol. 2025 Dec 4;16:1674145. doi: 10.3389/fpsyg.2025.1674145 (PMC12713118; doi:10.3389/fpsyg.2025.1674145)
Supplement: Supplementary file 3 [file Data_Sheet_3.DOCX]

**Survey on College Students’ Classroom Silence Behavior**

**Instructions**: This questionnaire is designed to understand the reasons behind students’ reluctance to ask questions or speak up in class. All responses are anonymous and for academic research only. Please answer based on your actual classroom experience.

**I. Classroom Silence Behavior (Please check the option that best represents your real thoughts.)**

| **No.** | **Statement** | **Strongly Disagree** | **Disagree** | **Neutral** | **Agree** | **Strongly Agree** |
| --- | --- | --- | --- | --- | --- | --- |
| Q1 | I usually don’t ask questions voluntarily in class. | ☐ | ☐ | ☐ | ☐ | ☐ |
| Q2 | Even if I have questions, I tend to remain silent. | ☐ | ☐ | ☐ | ☐ | ☐ |
| Q3 | I often avoid eye contact with the teacher during class questioning. | ☐ | ☐ | ☐ | ☐ | ☐ |
| Q4 | I tend to wait for others to speak before deciding to participate. | ☐ | ☐ | ☐ | ☐ | ☐ |
| Q5 | If the whole class is quiet, I’m more likely to stay silent too. | ☐ | ☐ | ☐ | ☐ | ☐ |

**II. Knowledge Conformity**

| **No.** | **Statement** | **Strongly Disagree** | **Disagree** | **Neutral** | **Agree** | **Strongly Agree** |
| --- | --- | --- | --- | --- | --- | --- |
| Q6 | If others don’t ask questions, I assume I understood correctly too. | ☐ | ☐ | ☐ | ☐ | ☐ |
| Q7 | I usually think there’s no need to speak if no one else is speaking. | ☐ | ☐ | ☐ | ☐ | ☐ |
| Q8 | When most classmates are silent, I start to doubt whether I should speak. | ☐ | ☐ | ☐ | ☐ | ☐ |
| Q9 | I don’t like to break the silence when everyone else is quiet. | ☐ | ☐ | ☐ | ☐ | ☐ |
| Q10 | I prefer to speak after someone else has spoken first. | ☐ | ☐ | ☐ | ☐ | ☐ |

**III. Social Anxiety and Fear**

| **No.** | **Statement** | **Strongly Disagree** | **Disagree** | **Neutral** | **Agree** | **Strongly Agree** |
| --- | --- | --- | --- | --- | --- | --- |
| Q11 | I often feel nervous or anxious before speaking in class. | ☐ | ☐ | ☐ | ☐ | ☐ |
| Q12 | I worry about being laughed at if I say something wrong. | ☐ | ☐ | ☐ | ☐ | ☐ |
| Q13 | I’m afraid of expressing my opinion in public settings. | ☐ | ☐ | ☐ | ☐ | ☐ |
| Q14 | I fear being negatively judged by the teacher or classmates for my questions. | ☐ | ☐ | ☐ | ☐ | ☐ |
| Q15 | My social anxiety affects my willingness to speak in class. | ☐ | ☐ | ☐ | ☐ | ☐ |

**IV. Perceived Classroom Atmosphere**

| **No.** | **Statement** | **Strongly Disagree** | **Disagree** | **Neutral** | **Agree** | **Strongly Agree** |
| --- | --- | --- | --- | --- | --- | --- |
| Q16 | My class encourages students to ask questions and engage in discussions. | ☐ | ☐ | ☐ | ☐ | ☐ |
| Q17 | I feel the relationships among classmates are harmonious, so I’m not afraid of being laughed at or rejected. | ☐ | ☐ | ☐ | ☐ | ☐ |
| Q18 | The teacher is generally supportive and encouraging toward students’ questions. | ☐ | ☐ | ☐ | ☐ | ☐ |
| Q19 | In my class, speaking up is viewed as a positive behavior. | ☐ | ☐ | ☐ | ☐ | ☐ |
| Q20 | The class discussion environment makes me more willing to express my thoughts. | ☐ | ☐ | ☐ | ☐ | ☐ |

**V. Self-Efficacy and Confidence in Expression**

| **No.** | **Statement** | **Strongly Disagree** | **Disagree** | **Neutral** | **Agree** | **Strongly Agree** |
| --- | --- | --- | --- | --- | --- | --- |
| Q21 | I believe I can ask valuable questions. | ☐ | ☐ | ☐ | ☐ | ☐ |
| Q22 | I am confident in my ability to express myself in class. | ☐ | ☐ | ☐ | ☐ | ☐ |
| Q23 | Even if my opinion differs from others, I dare to express it. | ☐ | ☐ | ☐ | ☐ | ☐ |
| Q24 | I believe my questions are helpful to my classmates. | ☐ | ☐ | ☐ | ☐ | ☐ |
| Q25 | I feel I can overcome the nervousness of speaking in class. | ☐ | ☐ | ☐ | ☐ | ☐ |
